# Faculty Development: From Program Design and Implementation to Scholarship

**DOI:** 10.3205/zma001126

**Published:** 2017-10-16

**Authors:** Yvonne Steinert

**Affiliations:** 1 McGill University, Faculty of Medicine, Centre for Medical Education, Richard and Sylvia Cruess Chair in Medical Education, Lady Meredith House, Montreal, Canada

## Commentary

This issue of the Journal is devoted to faculty development programs and activities. In a time of educational change and transformation, this publication underscores the faith of our institutions in faculty development and in the ongoing renewal of our faculty members. This issue also highlights an international phenomenon: despite a plethora of program descriptions of faculty development – and a growing emphasis on the evaluation of such programs – research in faculty development has not kept up with the proliferation of innovative and timely faculty development programs and activities. Based on previous systematic reviews of the faculty development literature to enhance teaching effectiveness [1], [2], we know that overall satisfaction with faculty development programs is generally high and that faculty members tend to report positive changes in attitudes, knowledge, skills and behaviors following a particular program or activity. We also know that changes in student learning and organizational practice are infrequently reported and that there is much that still needs to be understood. For example, little is known about how change occurs as a result of a “formal” faculty development activity, how professional development – and individual change (or growth) – unfolds in the workplace, and how faculty development interventions can influence the organization (or institution) in which these activities take place. Comprehensive program evaluation, rigorous research studies, and knowledge translation are vitally important in moving the field of faculty development forward, and we will focus on these three specific areas to help advance our collective objectives.

## Ensuring Comprehensive Program Evaluation

Most faculty development programs described in the literature (and in this issue) include an evaluative component. Some use Kirkpatrick and Kirkpatrick’s framework [3] to guide data collection; others primarily assess participant satisfaction and self-perceptions of learning. Irrespective of the evaluation methods used and the data collected, it is helpful to remember the goals of evaluation, which often fall into three distinct areas: demonstrating accountability, generating new insights and understanding, and supporting and guiding development [4]. Evaluation can also be formative, often known as “process evaluation”, or summative, often referred to as “outcomes evaluation” [5], and the process can target policy, curriculum content, or teaching and learning. In faculty development, it is important to conduct a comprehensive evaluation of the intervention (or program) from the outset, and not to add it as an afterthought, which is often the case. It is also recommended that we collect more than what has often been called “happiness” (or reaction) data and that we be systematic in our approach, following five key principles highlighted by the American Evaluation Association [http://www.eval.org/p/cm/ld/fid=51 retrieved December 2016]: systematic inquiry, competence, integrity, respect for people, and responsibility. Importantly, we should also collect the views and perspectives of multiple stakeholders and utilize the acquired information in a meaningful way. As Spencer [5] has suggested, program evaluation must be given the attention and methodological rigour that it requires; if this is not possible, resources may be wasted and inaccurate or irrelevant conclusions may be drawn. 

The potential overlap between program evaluation and research should also be considered, as comprehensive program evaluations can yield findings that have external as well as internal benefits. It has been stated that research aims to generate new knowledge and understanding, usually for consumption by the academic community, whereas, evaluation aims to provide useful feedback to inform and/or influence decision-making within a community of practice [5]. However, regardless of what the ultimate goal might be, evaluation must be as comprehensive as possible, asking relevant questions, using appropriate methods of data collection and analysis, assessing the perspectives of multiple stakeholders, adhering to ethical principles, and reporting (and disseminating) findings in a truthful manner [6]. At the same time, as with research, there is no one “correct” pathway to rigorous program evaluation, and a creative and flexible approach, informed by best evidence and practice, is recommended. 

## Conducting Rigorous Research Studies

Research in faculty development has been limited in both scope and methodology and needs to be more rigorous in order to advance the field [2]. 

### Expanding our Scope

With regard to expanding our scope, there are many potential areas of inquiry, a few of which will be explored here: analysing the process of “formal” (structured) faculty development programs and activities; understanding how health professions educators and teachers learn in the workplace; and evaluating context and organizational change. 

Although the need to assess faculty development outcomes and impact remains a priority, we must also carry out process-oriented studies to better understand how change occurs as a result of a particular faculty development intervention, irrespective of its format or approach [7]. As an example, we should consider expanding the focus of outcome-oriented studies to compare how different faculty development interventions (e.g. workshops or longitudinal programs) promote change in faculty members’ competence and performance [8]. We should also try to better dissect the “key features” of most faculty development interventions (e.g. experiential learning; peer support; feedback and reflection) to know which processes lead to change at the individual and group level. The assessment of change, within the individual (e.g. how did a clinical teacher’s attitudes and values change) and over time, would also be worthwhile, with a particular focus on transfer of training [9], the “durability” of change, and factors which help to sustain change [10]. Given health professionals’ roles in creating change at multiple levels, including educational, social and health care transformation, assessment over time is critical [7]. In addition, many of the outcomes expected in a planned faculty development program take time to emerge. This serves as a further impetus to promote longitudinal assessment and follow-up. 

We also need to remember that faculty development interventions are complex in nature. Pawson et al. [11] have described features of complex interventions that include the following: they are usually based on several hypotheses or theories, some more well-defined and/or evidence-based than others; they usually involve a wide range of participants (e.g. faculty developers; participants; learners); and they may require a “long journey” (from design to delivery), with success dependent on a cumulative chain of events that is usually non-linear, with multiple pathways and feedback loops. In addition, complex interventions are embedded in multiple social systems, and it is important to consider this complexity when evaluating – or studying – faculty development programs or activities. As Cook et al. [6] have suggested, we should pursue “clarification” studies (in addition to “description” and “justification” studies) to deepen our understanding and advance the art and science of medical education. This recommendation, to understand “why” or “how” something works, is particularly timely in this context as well.

As described in the literature, health professionals learn about their faculty roles in both formal and informal ways [11]. However, although “there are strong indicators that a great deal of learning takes place in the workplace, relatively little appears to be known about how people learn informally or about the relative value of different types of learning experiences” [12]. Even less is known about how health professionals learn in the workplace, even though this is where their educational roles first emerge. Clarke and Hollingsworth [13] have argued that it is time to shift our thinking away from programs that “change teachers” to viewing faculty members as “active learners shaping their own professional growth through reflective participation in professional development programs and practice”. This perspective, together with that suggested by O’Sullivan and Irby [14], [15] and outlined below, provides a research agenda for the future. It also underscores the need to understand the value of role modelling, reflection and engagement in workplace learning [16] as well as the benefit of exploring the following questions: Have we created a false dichotomy between work and learning? What is the value of participation in work as a catalyst for learning? How can we make workplace learning more visible? 

Workplace learning is closely tied to the notion of communities of practice [17], and a number of authors [15], [18], [19] have recommended that future research in this area should explore current understandings of communities of practice, with attention to how they evolve, how they function, and how they can lead to individual and organizational growth and development. Such research would also be helpful in illuminating how communities of health professions educators can be developed and sustained [7]. At the same time, we should think about how workplace learning and communities of practice can lead to enhanced competencies for faculty members. 

As discussed above, and as is evident in this issue of the journal, faculty development occurs in a complex environment in which many unforeseen and unpredictable variables play a role. As a result, we should try to conduct more research that tries to understand the contextual and organizational factors that can promote or hinder the professional development and learning of faculty members. Looking at faculty development across sites and across cultures (e.g. national cultures; organizational cultures; professional cultures) would enhance our understanding of the influence of context on these activities. We also need to further assess and understand the impact of faculty development on the organization. A number of authors have stated that faculty development can – and should – enhance organizational capacity [7], [20]. However, we need to move beyond anecdotal observations and verify whether this assertion is, in fact, true. The paucity of research assessing the impact of faculty development on the organization is surprising [1], [8]. As a result, there is a clear need to assess outcomes and impact at the organizational and systems level. Research in this area would also provide valuable insights to help guide future policies and practices.

#### Refining our Methodology

From a methodological point of view, studies in this field have been limited by a number of challenges. For example, the majority of faculty development studies have used descriptive, single-group designs to examine outcomes, which make it difficult to attribute outcomes directly to interventions [8]. Many studies also rely entirely on post-intervention measures or collect data several years after the intervention took place [1], [2], making the “attribution of change” equally challenging. In addition, researchers have tended to over-emphasize a positivist paradigm and to under-utilize qualitative methodologies which, in many ways, can more easily capture the process of change. However, the need for research in this field has never been greater, as we try to promote academic inquiry and scholarship, inform “best” practices, and remain responsive to organizational needs and priorities. 

To move the research agenda forward, it would be helpful to go beyond a positivist paradigm and conduct research studies framed by post-positivist, interpretivist and critical theory research paradigms [7], using associated methodologies to enrich our understanding of faculty development. As O’Sullivan and Irby [15] have stated, “a paradigm defines the prevailing model of exemplary practices for a community of researchers; it illuminates areas for investigation and obscures others”. Changing paradigms would enable new perspectives and encourage us to consider innovative conceptual approaches and methodologies. 

We also need to ensure that research in this field is informed by theoretical models or conceptual frameworks. As an example, O’Sullivan and Irby [14], [15] have suggested that we use a conceptual framework that incorporates the notion of a faculty development community and a workplace community to conduct research in this area. More specifically, in this model, the faculty development community includes the participants involved in faculty development, the programs (i.e. curricula) offered, the facilitators of faculty development initiatives, and the context in which faculty development is organized (e.g. classroom or clinic). The workplace community is equally important, as participants involved in faculty development collaborate with other teachers or staff members, have relationships and networks in the workplace, fulfill tasks and activities within the educational program, have mentors and coaches in the work setting, and work in an organizational context characterized by a culture that either supports or inhibits educational change. Not surprisingly, these authors postulate that research in this area should focus on process and outcomes, including relationships within the faculty development program and the workplace.

Clearly, many theoretical models are available to guide our work [16], [17], [19]. However, irrespective of which conceptual approach we adopt, we should strive to utilize theory in the design of our research and in the interpretation of our results. We should also consider incorporating new methodologies and methods. In many ways, we need to move away from an over-reliance on experimental and quasi-experimental designs and consider qualitative designs, using phenomenology, ethnography, case studies and mixed methods [8], [21]. We should also consider using diverse methodologies suggested by O’Sullivan and Irby [15]. For example, educational design research is pertinent to faculty development because it enables the researcher to attend to program goals and design, a description of how the intervention unfolds, achieved outcomes and lessons learned [22]. Success cases, which can be used concurrently with design research, fit within an interpretivist paradigm and aim to reveal how an initiative is working and what contextual factors support successful implementation [23]. Sustainability narratives, which are considered to be a research methodology that lies “outside the normal modes of inquiry for the education community”, explores the development of a society through the lens of human and environmental systems and imagines what the future would be like if people’s lives were improved [24]. The use of this methodology and that of narrative research [25] would enable a rich understanding of the faculty development process as well as individual and organizational change. 

Much has also been written about the need to improve the research methods used in this field of inquiry [1], [2], [8]. Suffice it to say that we should use validated outcome measures, including newer methods of behavioural or performance-based measures of change, as well as multiple methods and data sources to assess process and outcome. To date, we have witnessed an over-reliance on self-assessment methods and survey questionnaires to assess change. Moving forward, we should consider the use of alternative data sources and try to ascertain as many stakeholder perspectives (e.g. students; colleagues) as possible. Lastly, irrespective of the methodologies chosen, we should ensure congruence between study design, research questions and the methodology that we choose. 

## Promoting Knowledge Translation

No discussion of research on faculty development in the health professions (knowledge creation) would be complete without talking about how our work in this area can inform our practice (knowledge-to-action), as we must ensure that research informs practice – and that practice informs research [26]. Knowledge translation in this field remains an urgent priority from two perspectives. On the one hand, faculty development can be perceived as an important means of knowledge translation as we take the available evidence and “translate” it into action for our colleagues. Interestingly, as Graham et al. [27] have summarized, there are seven stages in moving knowledge into practice that include: identifying a problem in practice, or a gap in knowledge, and identifying, reviewing, and selecting the knowledge to be implemented to address the gap; adapting or customizing the knowledge to the local context; evaluating the determinants of the knowledge use; selecting, tailoring and implementing interventions to address the knowledge or practice gap; monitoring the knowledge use in practice; evaluating the outcomes or impact of using the new knowledge; and determining strategies for ensuring that the new knowledge is sustained. These diverse steps are particularly appropriate to the design and delivery of a variety of faculty development interventions, even though they are rarely examined from this perspective. 

Knowledge translation, or implementation science, is also important in this field as we have an obligation to not only evaluate or study our faculty development interventions, but also to disseminate our findings and help to ensure that faculty development practices are informed by the “best” available evidence. In different ways, faculty development can be seen as a form of scholarship that includes discovery, integration, application, and teaching [28]. As an example, the scholarship of teaching, which is made possible through discovery, application and integration, entails two components. It refers to the application of scholarly activities to teaching as well as the effective dissemination of results. As Glassick [29] has stated, “teaching becomes scholarship when it is made public, is available for peer review and critique, and can be reproduced and built on by other scholars”. This notion of scholarship is particularly pertinent to faculty development and to this issue of the journal, which aims to bring together educators and scholars as they design, implement, and evaluate faculty development initiatives across the educational spectrum. 

## Competing interests

The author declares that she has no competing interests. 
